# Modulation of macrophage polarization and lung cancer cell stemness by MUC1 and development of a related small-molecule inhibitor pterostilbene

**DOI:** 10.18632/oncotarget.8101

**Published:** 2016-06-04

**Authors:** Wen-Chien Huang, Mei-Lin Chan, Ming-Jen Chen, Tung-Hu Tsai, Yu-Jen Chen

**Affiliations:** ^1^ MacKay Medical College, Taipei, Taiwan; ^2^ Department of Surgery, Division of Thoracic Surgery, MacKay Memorial Hospital, Taipei, Taiwan; ^3^ Department of Radiation Oncology, MacKay Memorial Hospital, Taipei, Taiwan; ^4^ Institute of Traditional Medicine, School of Medicine, National Yang Ming University, Taipei, Taiwan; ^5^ Department of Surgery, Division of Colorectal Surgery, MacKay Memorial Hospital, Taipei, Taiwan; ^6^ Department of Education and Research, Taipei City Hospital, Taipei, Taiwan; ^7^ College of Chinese Medicine, China Medical University, Taichung, Taiwan

**Keywords:** tumor-associated macrophages (TAMs), lung cancer stem cells (CSCs), MUC1, pterostilbene, M2 polarization, Immunology and Microbiology Section, Immune response, Immunity

## Abstract

Tumor-associated macrophages (TAMs) polarized to the M2 phenotype play key roles in tumor progression in different cancer types, including lung cancer. MUC1 expression in various types of cancer is an indicator of poorer prognosis. Elevated MUC1 expression has been reported in inflammatory lung macrophages and is associated with lung cancer development. Here, we investigated the role of M2-polarized TAMs (M2-TAMs) in the generation of lung cancer stem cells (LCSCs) and tested pterostilbene, a small-molecule agent that modulates MUC1 expression in lung cancer cells, with the goal of subverting the microenvironment toward a favorable anti-tumor impact. We found that MUC1 was overexpressed in lung cancer patients, which was associated with poor survival rates. M2-TAMs and cancer cell lines were co-cultured in an experimental tumor microenvironment model. The expression levels of MUC1 and cancer stemness genes significantly increased in lung cancer cells in the presence of the M2-TAM cells. Intriguingly, pterostilbene dose-dependently suppressed self-renewal ability in M2-TAMs-co-cultured lung cancer cells, and this suppression was accompanied by downregulation of MUC1, NF-κB, CD133, β-catenin, and Sox2 expression. Moreover, MUC1-silenced M2-TAMs exhibited a significantly lower ability to promote LCSC generation and decreased levels of NF-κB, CD133, and Sox2. The results suggest that MUC1 plays an important role in TAM-induced LCSC progression. Pterostilbene may have therapeutic potential for modulating the unfavorable effects of TAMs in lung cancer progression.

## INTRODUCTION

Tumors are a heterogeneous biological system whose complexity may even exceed normal somatic tissues. This view is supported by the finding of a variety of different cell types within the tumor microenvironment where tumor cells and the surrounding stromal cells interact and evolve in a dynamic fashion during the course of multistep tumorigenesis. In fact, the tumor microenvironment has been regarded as a major hallmark of cancer [1]. One of the major stromal cell types in a variety of different cancers is the tumor-associated macrophage (TAM). It has been reported that the invasive fronts of advanced carcinomas commonly contain a high number of TAMs [2]. TAMs play a role in Type 2 reactions, inducing inflammatory responses and affecting adaptive immunity. They stimulate cell proliferation by producing pro-inflammatory cytokines and enhance tissue remodeling, angiogenesis, and metastasis. Hence, TAMs directly modulate the physiological and biochemical processes within the tumor microenvironment, enhancing metastatic potential and resistance to treatments [2]. Both clinical and *in vivo* studies have established a strong positive correlation between the TAM density in a tumor specimen and poor clinical prognosis [3, 4]. For instance, systemic depletion of M2-macrophages decreases the formation of lung metastasis [5]. Notably, M2-macrophages produce multiple growth factors (such as EGF, FGF, HGF, PDGF, and TGFβ) and pro-inflammatory cytokines (including interleukin-1, interleukin-6, interferons, and TNFα) to stimulate the growth, migration, and invasiveness of cancer cells. TAMs also assist cellular invasion by producing a spectrum of proteases, including uPA and various matrix metalloproteinases, to degrade the extracellular matrix [4]. Recent studies have shown that the invasiveness and drug resistance of tumor cells were significantly enhanced when the cells were co-cultured with macrophages or exposed to macrophage conditioned medium. This phenomenon has been attributed to the secretion of TNFα from macrophages, because antibody neutralization of TNFα significantly suppressed macrophage-mediated tumor cell invasion [6, 7]. Thus, TAMs, the main source of inflammatory signals in the stroma of the tumor microenvironment, could be an important drugable target.

The presence of cancer stem cells (CSCs) has been reported in a diverse variety of cancer types including in the lung and these cells contribute to treatment failure, distant metastases, and disease recurrence [8]. CSCs have been characterized as containing many attributes of normal stem cells including self-renewal ability, resistance to stress, pluripotency, and the potential to transition between epithelial and mesenchymal statuses [9]. The generation and maintenance of CSCs involve extremely complex molecular signaling networks that remain largely unclarified. However, the local tumor microenvironment has been shown to play an influential role. TAMs appear to create an inflammatory environment which promotes epithelial-to-mesenchymal transition (EMT) which subsequently leads to the generation of CSCs [10]. Recent evidence demonstrates that the expression of pro-oncogenic mucin, MUC1 is elevated in response to inflammation in airway epithelial cells and is associated with lung cancer development. The increased MUC1 expression appears to activate signal-regulated kinase (ERK) mediated phosphorylation and the CCAAT/enhancer-binding protein β (C/EBPβ) transcription factor in breast cancer cells. MUC1 is produced by inflammatory lung macrophages which subsequently secrete TNFα and promote cancer progression [11, 12]. Based on these premises, we hypothesized that MUC1 secretion by M2-TAMs may be a key step in generating CSCs in the lung.

In this study we set out to examine the role of MUC1 in TAMs and its association with the generation of lung cancer stem cells. Essentially, in the presence of M2-TAMs, non-small cancer cells demonstrated an increased percentage of CD133^+^ cells accompanied with increased stemness and inflammation-associated genes including CD133, Sox2, and NF-κB. Muc1 silencing in TAM precursor cells decreased TAM's ability to promote the generation of CD133^+^ CSCs and suppressed stemness and inflammation pathways. We found that the treatment of phytochemical pterostilbene led to the suppression of TAM-mediated CSC generation *via* modulating MUC1 signaling. In conclusion, we have shown that MUC1 was involved in TAM-mediated lung CSC generation and agents such as phytochemical pterostilbene that can negatively modulate MUC1 expression could potentially be used to suppress lung CSC generation and CSC-associated malignancy.

## RESULTS

### Muc-1 is overexpressed in non-small cell lung cancer (NSCLC) patients and correlates with a poor prognosis

**Figure 1:**
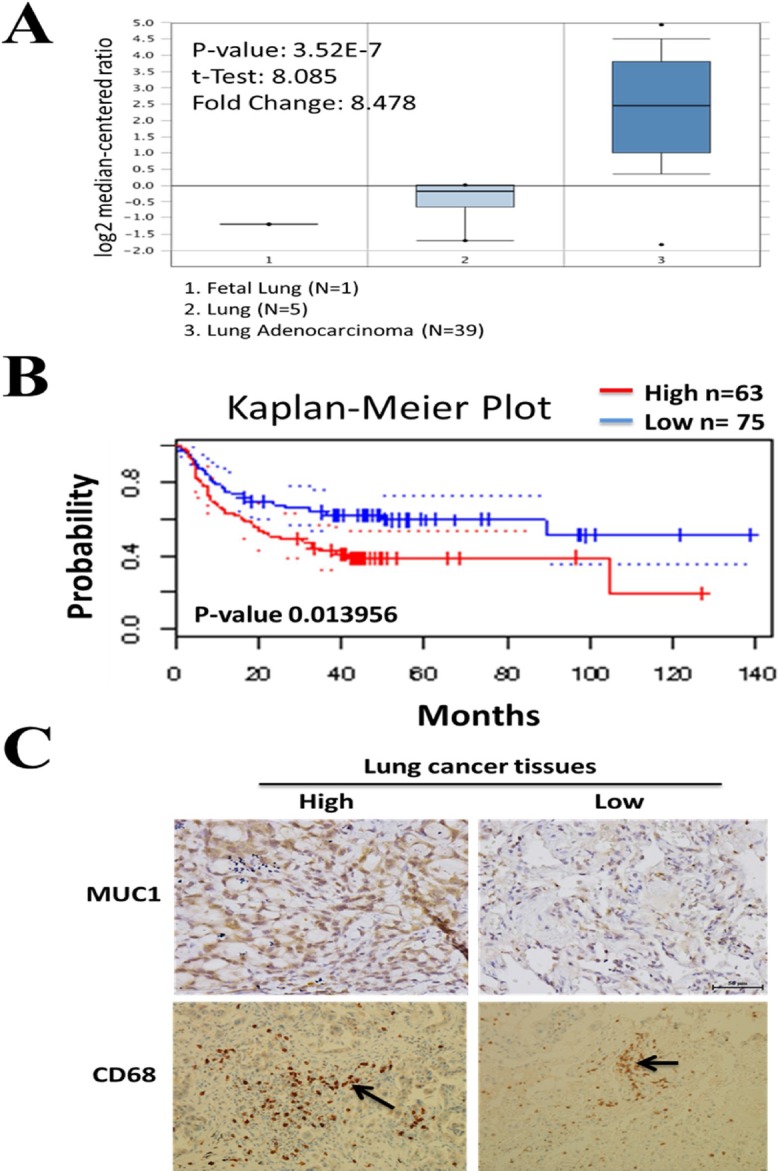
Clinical association of Muc-1 in lung cancer patients **A.** Using Oncomine database search, we found a significant increased level of Muc-1 (8.478-fold increase) was detected in lung adenocarcinoma (*N* = 39) as compared to normal lung tissues such as fetal lung (*N* = 1) and adult lung samples (*N* = 5). Garber lung Database [12] was selected. **B.** PrognoScan analysis indicated that lung cancer patients with high Muc-1 expression (*n* = 63) were associated with a poor overall survival rate as compared to patients with lower or no Muc-1 expression (*n* = 75). **C.** Representative immunohistochemistry photomicrographs of MUC1 (cytoplasmic) or macrophage marker CD68 expression in NSCLC. Arrow indicates CD68^+^ TAM.

Using a database in the public domain (Garber lung Database [12], Oncomine, Figure 1A), we identified an approximately 8.5-fold increase of MUC1 transcript in patients with lung adenocarcinoma (*N* = 39) as compared to normal lung tissues, including fetal lung (*N* = 1) and adult lung samples (*N* = 5). More importantly, using the PrognoScan software and database, a high MUC1 expression level (*n* = 63) was found to correlate with a significantly poorer overall survival for patients with lung cancer, compared to patients with lower MUC1 expression (*n* = 75) (Figure 1B). Paraffin-embedded sections of clinical NSCLC specimens revealed strong staining of MUC1 and, by contrast, weak staining in the normal lung counterparts (Figure 1C). We tabulated that 65% of tumor specimens demonstrated high expression of MUC1, whereas 35% displayed a relatively lower MUC1 expression. Subsequently, we examined the potential role of MUC1 within the tumor microenvironment by analyzing the number of infiltrating TAMs. Notably, the count was significantly higher in the lung tumor specimens than in normal lung tissue. More specifically, TAMs with positive CD68 staining were in a significantly higher number of the NSCLC tissues that also stained strongly with MUC1. We tabulated that approximately 32 (64%) of the cases of NSCLC specimens were both MUC1 and CD68 positive (Table 1).

**Table 1:** Expression of MUC1 and CD68 in lung adenocarcinoma specimens

	MUC1 (%)	CD68 (%)	MUC1 (%) and CD68(%)
Positive	32(65%)	35(70%)	32(64%)
Negative	18(35%)	15(30%)	18(26%)

### M2-polarized tumor-associated macrophages promoted lung cancer stemness in NSCLCs during co-incubation

**Figure 2:**
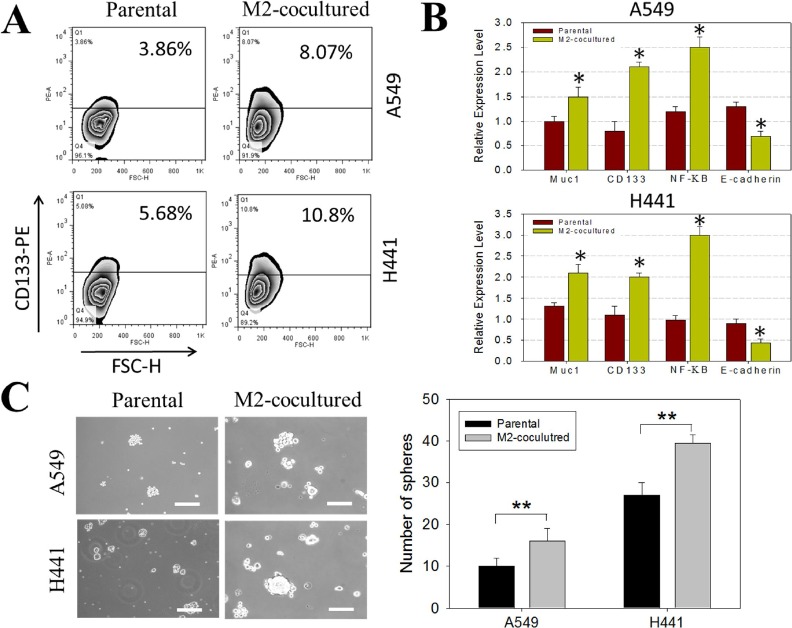
M2 TAM co-incubation leads to increase of CD133M^+^ lung cancer stem-like cells **A.** FACS analysis demonstrates that the CD133^+^ cell population increased in both A549 (from 3.86 to 8.07%) and H441 (from 5.68 to 10.80%) in the presence of M2 TAM. **B.** Gene profile was also changed in the presence of M2 TAMs. MUC1, Sox2 (stemness), NF-κB (inflammation) expression level were increased in the presence of M2 TAMs while a major EMT marker, E-cadherin was downregulated. **C.** Sphere forming assay of sphere-forming assay of A549 and H441 cells in the presence of M2 TAM. Cells (1×10^3^cells/ml) were cultured in serum-free DMEM/F12 medium with growth factors (10 ng/ml of EGF and bFGF each) for 10 days and counted the spheres as described in ‘Materials and methods’. Representative images of spheres (left magnification, 100x) and quantification of the assay (right).

M2-polarized tumor-associated macrophages (M2-TAMs) have been shown to promote the progression of lung cancer [13, 14]. However, the role of M2-TAMs in the production of lung cancer stem cells has not been fully elucidated. Here we co-incubated M2-TAMs with NSCLC cell lines, A549 and H441. We used flow cytometric analysis to observe an increase in the CD133 positive cell populations in both cell lines (approximately 8.07% and 10.8% in A549 and H441, respectively) after co-incubation with M2-TAMs (Figure 2A). On the mRNA level, after co-incubation with M2-TAMs, Muc-1, CD133 (stemness), and NF-κB (inflammation) were up-regulated, while E-cadherin (epithelial marker) was down-regulated (Figure 2B). More importantly, in the presence of M2-TAMs, NSCLC cells exhibited significantly enhanced self-renewal ability as represented by an increased number of tumor spheres formed in both A549 and H441 lines (Figure 2C). These data suggested that M2-TAMs promoted the generation of lung cancer stem cells.

### Treatment of NSCLC cell lines with pterostilbene reduced the induction of stemness by M2-TAMs

**Figure 3:**
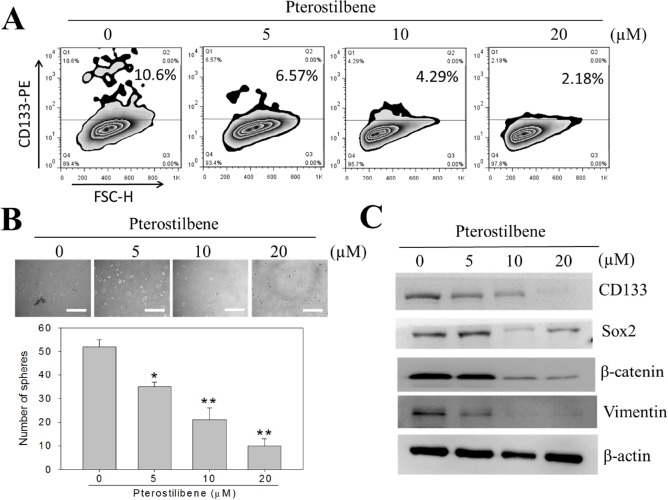
Treatment of pterostilbene decreased the percentage of CD133^+^ H441 cells co-cultured with M2 TAMs **A.** M2-co-cultured H441 cells were treated with different concentrations of pterostilbene and analyzed for the abundance of CD133^+^ cells flow cytometrically. Dose-dependently, pterostilbene decreased the percentage of CD133^+^ H441 cells. **B.** Pterostilbene suppressed the tumor sphere-forming ability of CD133^+^ H441 cells in a dose-dependent fashion. **C.** Western blots demonstrated that pterostilbene treated H441 tumor spheres demonstrated a decreased level of CD133, Sox2 (both stemness markers), β-catenin and epithelial-mesenchymal transition (EMT) key marker, Vimentin in a dose-dependent manner.

Next, we examined the potential effects of pterostilbene in decreasing stemness in NSCLC cell lines. Pterostilbene suppressed the percentage of CD133 positive H441 cells dose-dependently when cells were co-cultured with M2-TAMs, as demonstrated by our flow cytometric analysis (Figure 3A). Consistently, pterostilbene treatment resulted in a dose-dependent decrease in self-renewal ability, as manifested in decreased numbers of tumor spheres formed (Figure 3B). For instance, approximately a 30% decrease in the number of tumor spheres formed was observed at the highest dosage (20μM). Decreased stemness and self-renewal ability were accompanied by the decreased expression of key molecules such as CD133, Sox2, and β-catenin. Notably, Vimentin expression was also negatively regulated by treatment with pterostilbene (Figure 3C). We systematically examined the effects of pterostilbene on different human lung cancer cells (Supplemental Figure S1). PC-9 (EGFR mutation, in-frame deletion in exon 19) and PC-9/GR (gefitinib-acquired resistant lung cancer cells) cell lines were cultured in medium containing increasing concentrations of pterostilbene. Cell viability assays indicated that the IC50 values of pterostilbene were approximately 2- to 5-fold lower than those of resveratrol in both lung cancer cell lines (data not shown). Subsequently, we assessed whether a combined treatment of pterostilbene and cisplatin could lead to greater apoptosis. We first measured the caspase-3 activity in NSCLC H441 cells that had been treated with selected dosages. We found that the combined treatment induced a significant increase in caspase-3 activity, accompanied by an increased percentage of annexin V positive H441 cells, as assessed by flow cytometric analysis (Supplementary Figure S2). Our results suggested that pterostilbene alone is a more potent apoptosis inducer than resveratrol in lung cancer cells. The enhanced apoptosis-inducing ability of pterostilbene may be attributed to its superior bioavailability.

### Pterostilbene treatment prevented M2-TAM polarization and decreased side-population cells in NSCLC

**Figure 4:**
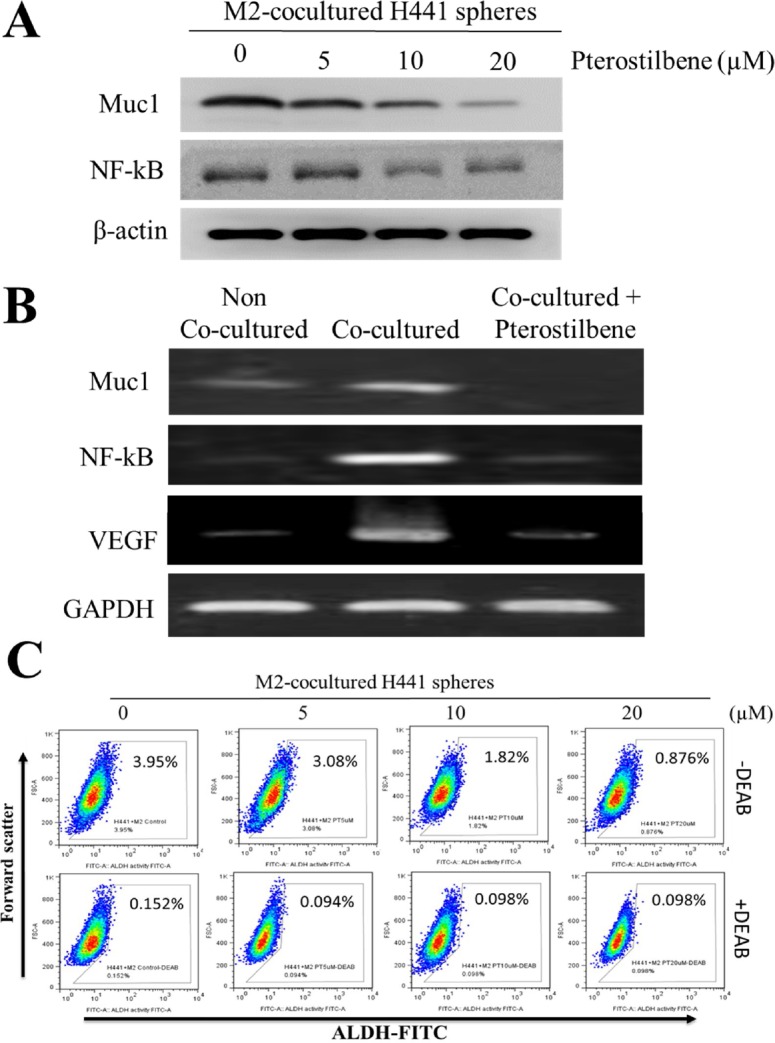
Pterostilbene treatment prevented M2 polarization *via* down-regulation of Muc1/NF-κB signaling axis **A.** Pterostilbene treatment suppressed the Muc1 and NF-κB expression in M2 polarization of THP-1 cells. **B**. Muc1, NF-κB and VEGF mRNA expression levels in M2 polarization of THP-1 cells were significantly decreased after co-culturing with pterostilbene in H441 cells. **C**. FACS analysis indicated that in the presence of pterostilbene, the percentage of ALDH subpopulation cells in TAM co-cultured H441 was significantly decreased.

We have established that pterostilbene treatment decreased CD133 positive cells in both NSCLC lines even in the presence of M2-TAMs. We then examined the possibility that pterostilbene treatment could prevent M2-TAM polarization. We demonstrated that pterostilbene treatment dose-dependently decreased MUC1 expression, along with NF-κB (Figure 4A), in M2-co-cultured H441 spheres. NF-κB has been established as a pro-inflammatory marker as well as a key molecule in promoting the M2-TAM phenotype [15, 16]. In order to determine whether MUC-1 is involved in M2-TAM polarization, THP-1 monocytes were pretreated with pterostilbene and the expression of M2-biomarkers was profiled. It was observed that the mRNA levels of MUC-1, NF-κB, and VEGF in the THP-1-H441 co-culture system was significantly decreased after pterostilbene treatment as compared with the untreated control group. This finding suggests that pre-treatment with pterostilbene abolished the M2-macrophage marker expression in the THP-1 cells that would have been promoted by MUC-1 in the absence of treatment (Figure 4B). Our results suggested that H441-secreted MUC-1 could promote M2-TAM polarization and alter the pro-inflammatory cytokine expression pattern. The involvement of M2-TAMs in a tumor microenvironment may facilitate the escape of cancer cells from immune surveillance and their subsequent progression. In addition, the treatment of pterostilbene dose-dependently decreased the ALDH+ subpopulation of cells in TAM-co-cultured H441 spheres (Figure 4C). These data suggest that pterostilbene treatment prevents M2-TAM polarization, directly or indirectly leading to reduced numbers of ALDH+ lung cancer stem-like cells.

### Decreased Muc-1 expression in M2-TAMs correlates with a decreased ability to promote stemness in NSCLC

**Figure 5:**
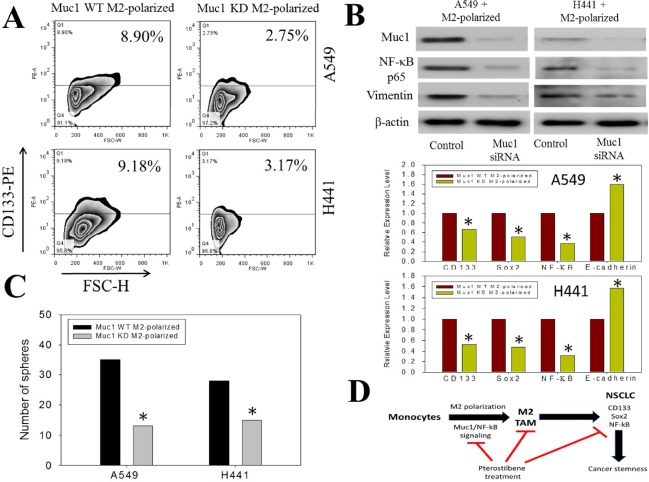
Decreased stemness-promoting ability in Muc1 silenced M2 TAMs **A.** A549 and H441 cells were co-incubated with wild-type (WT) M2 TAMs and Muc1-silenced (KD) TAMs and analyzed for their stemness. FACS results demonstrated that the percentage of CD133^+^ cells did significant increase as in ones co-incubated with the wild-type M2 TAMs. **B.** Western blots of A549+M2 and H441+M2 cells treated with Muc1 siRNA demonstrated that the expression levels of p65 (NF-κB) and vimentin were downregulated as compared to that in the cells that received control siRNA. Comparative gene profiling of NSCLC cells co-incubated with wildtype (WT) and Muc1 knock-down (KD) TAMs. Data indicated that Muc1-silenced TAMs did not induced stemness in A549 and H441 cells. CD133, Sox2, NF-κB expression level was comparatively lower in the Muc1 KD groups as compared to those in WT counterparts. Gene expression analysis of selected M1 and M2-associated transcripts in macrophages after being treated with control siRNA or MUC1 siRNA in THP-1 cells. **C.** Self-renewal ability was compromised in Muc1 KD M2 co-cultured NSCLCs. Both A549 and H441 showed decreased tumor sphere forming ability when co-incubated with Muc1 KD TAMs. **D.** Proposed mechanistic actions of Muc1 in M2 polarization and promoting stemness in NSCLCs.

After establishing the link between decreased levels of MUC-1 and NF-κB in M2-TAM polarization following pterostilbene treatment, we sought to further elucidate MUC-1′s role in promoting tumorigenesis and cancer stemness in NSCLC by silencing Muc1 expression in THP-1 cells. We found that cells with decreased MUC-1 could not promote the enrichment of CD133 positive cells under the same co-culture system described previously whereas wildtype M2-TAMs promoted the emergence of CD133 positive NSCLC cells (Figure 5A). To further confirm the functional relevance of MUC-1 in NF-κB-mediated signaling in M2-TAM-induced cancer malignancy, we used silencing techniques to inhibit Muc1 expression in M2-TAM-co-cultured A549 and H441 cells. We demonstrated that expression of NF-κB and the EMT markers in these cells were inhibited, evidenced by the suppression of NF-κB, p65, and vimentin. In agreement, our q-PCR analysis (Figure 5B) confirmed that MUC-1-silenced TAMs were unable to induce an elevation in CD133, Sox2 (stemness markers), or NF-κB (inflammation marker) expression but did elicit the expression of E-cadherin (epithelial marker). Consistently, Muc1-silenced TAMs could not enhance self-renewal ability, as reflected by a lower number of tumor spheres formed (Figure 5C). This indicates that MUC-1 plays an important role in regulating the M2-TAM phenotype and its participation in promoting stemness while acting as a stromal support to NSCLC cells. Taken together, our data indicate that pterostilbene inhibited lung metastasis and/or suppression of lung cancer stem cells *via* the downregulation of NF-κB, p65 signaling, and cancer stemness activation, acting through M2-TAMs (Figure 5D)

## DISCUSSION

Tumor-associated macrophages (TAMs) in the tumor microenvironment have been shown to play a critical role in promoting inflammation, proliferation, immune editing, and epithelial-to-mesenchymal transition (EMT) and subsequent metastasis [2, 4]. More importantly, cells with enhanced EMT potential have been shown to be positively correlated to the acquisition of a cancer stem cell phenotype [10]. Thus, targeting TAM-mediated signaling may represent an alternative and more effective therapeutic intervention. Recent studies have shown that MUC1 from TAMs activated the key inflammatory modulator, NF-κB, and contributed to lung cancer development [11, 17, 18]. MUC1 itself is an oncoprotein and has been shown to be elevated in cancer cells; MUC1 secreted from TAMs could act to enhance tumorigenesis. Our data were in agreement that in the presence of TAMs, NF-κB and MUC1 expression were increased in both A549 and H441 lung adenocarcinoma cell lines. In addition, the increased MUC1 expression was accompanied by increased expression of CD133 and decreased expression in E-cadherin. More importantly, the co-incubation of lung cancer cells with TAMs led to an increased self-renewal ability of the cancer cells. These findings were in agreement with others' studies that found that co-incubation with TAMs increased the CSC population in liver and breast cancer [7, 19].

Phytochemicals have been studied primarily for their effectiveness in treating various types of disorders, including cancer. Phytochemicals hold promising potential for anti-cancer drug development due to their abundance and their natural properties. Stilbenoids, belonging to the family of phenylpropanoids, have been found to exhibit many health benefits. For instance, resveratrol isolated from grapes has been extensively studied for its anti-inflammatory and anti-cancer properties [20, 21]. Pterostilbene, a double-methylated version of resveratrol has been shown to possess a higher bioavailability and stability than resveratrol [22]. More importantly, pterostilbene has been shown to suppress the enrichment of CD133-positive liver cancer stem cells *via* modulating NF-κB and its associated pathways [19]. This finding further supports the belief that NF-κB and inflammation contribute to the emergence of CSCs and the theory that agents that can negatively modulate this pathway may be of anti-CSC potential. In agreement, we showed that the treatment of pterostilbene dose-dependently suppressed the percentage of CD133^+^ lung cancer cells in the presence of TAMs and that this effect was mediated by the concomitant downregulation of MUC1, NF-κB, β-catenin, Sox2, and CD133. More importantly, the self-renewal ability of TAM-co-cultured lung cancer cells was significantly impaired when treated with pterostilbene. Importantly, pterostilbene has also been reported to disrupt β-catenin signaling and to inhibit colon tumorigenesis *in vivo* [23].

Pterostilbene is an analog of resveratrol, which has been implicated as a chemopreventive and therapeutic phytochemical for human lung cancer [24]. Pterostilbene contains one hydroxyl group and two methoxy groups in comparison to resveratrol, which has hydroxyl groups at those positions. The two methoxy groups are thought to increase the lipophilicity and oral absorption of pterostilbene, resulting in a higher bioavailability as compared to the non-methylated resveratrol [25]. A previous study reported that pterostilbene has an approximate bioavailability of 95%, compared to 20% for resveratrol [26]. The half-life of pterostilbene was shown to be around seven times longer than resveratrol [27]. Notably, pterostilbene appeared to be able to penetrate the blood-brain-barrier (BBB); it was detected in the brain tissue homogenates of mice following oral administration [28, 29]. Moreover, no apparent adverse effects were observed under different dosing regimens for treating prostate cancer xenograft-bearing mice [28].

Equally important, pterostilbene not only acts on tumor cells but also on macrophages. Pterostilbene treatment has been shown to reduce LPS-induced nuclear translocation of the NF-κB subunit and its transcriptional activity in mouse macrophages [30]. Consistently, tumor-associated macrophages were demonstrated to promote metastasis in epithelial cancer cells *via* the activation of NF-κB and JNK signaling [3]. Together, these studies and ours suggested that pterostilbene negatively modulated tumorigenesis *via* downregulating the NF-κB pathway. However, our data provided an additional link to this TAM-mediated lung tumorigenesis. MUC1, an oncoprotein that has been shown to be elevated in many cancer types including the lung, also is elevated in macrophages. We found that MUC1 expression was elevated in lung tumor spheres and pterostilbene treatment led to decreased expression of MUC1 and NF-κB, leading strong support for the development of pterostilbene as an anti-CSC agent.

To delineate MUC1′s role in CSC generation and its relationship with TAMs, we silenced MUC1 in THP-1 cells. We found that MUC1-silenced THP-1 cells could not increase the percentage of CD133^+^ lung cancer cell populations, unlike their wildtype counterparts, suggesting that MUC1 may be involved in promoting M2-TAM polarization. In support, pterostilbene-treated THP-1 cells showed decreased MUC1 protein levels accompanied by decreased M2-TAM markers CD68^+^/CD163^high^ whereas M1 markers CD68^+^/CD80^high^ increased. This is in accordance with a number of studies where an increased number of M2 polarized TAMs is associated with poorer prognosis in cancer patients [31–34]. More importantly, a recent study reported that activated macrophages induce TNF-alpha secretion through PPAR-gamma, ERK, and MUC1 signaling and contribute to smoke-induced lung cancer, establishing a direct involvement of MUC1 in TAM and lung cancer [13]. However, it remains to be explored how MUC1 directly contributes to the generation of lung CSCs. We hypothesized that MUC1 activation in both lung cancer cells and TAMs may trigger TNF-α mediated signaling events that promote CSC generation. Interestingly, TNF-α signaling has been shown to be involved in cancer progression and survival of chronic myeloid leukemia [35]. A similar signaling network may be present in lung cancer. Further investigations are underway in our laboratory to examine this possibility.

Collectively, we have shown that the presence of M2-TAMs promotes the generation of LCSCs through the upregulation of stemness genes that are associated with stem cells and inflammation. Our data indicated that pterostilbene inhibited M2-polarization *via* suppression of MUC1 and suppressed the production of LCSCs, which would usually be induced by the presence of M2-TAMs. Therefore, further exploration of the clinical usage of pterostilbene, in combination with current interventions, should be considered.

## MATERIALS AND METHODS

### Chemicals

Pterostilbene (3,5-dimethoxy-4-hydroxystilbene), a synthetic compound initially found in plants, was purchased from Sigma-Aldrich (USA). Pterostilbene was dissolved in dimethyl sulfoxide (DMSO) and further diluted in sterile culture medium immediately prior to use. The purity of pterostilbene was determined by high-performance liquid chromatography (HPLC) as > 97%. MTT dye [tetrazolium dye (thiazolyl Blue tetrazolium bromide)] was purchased from Sigma-Aldrich (St. Louis, MO, USA). Primary antibodies to CD133, NF-κB (p65), SOX2, Vimentin, E-cadherin, and β-actin were purchased from Cell Signaling Technology (Boston, MA, USA). A TRIzol RNA isolation kit was obtained from Life Technologies (Rockville, MD, USA). Primers for RT-PCR, dNTP, reverse transcriptase and Taq polymerase were obtained from Gibco BRL (Cergy Pontoise, France). All other chemicals were of the highest pure grade available.

### Cell lines and culture

Two human lung cancer cell lines, A549 and H441, characterized as high metastatic phenotypes, as well as THP-1 cells were purchased from the American Type Culture Collection (Manassas, VA, USA). Lung cancer cells were maintained as monolayers in Dulbecco's modified Eagle's medium (DMEM, Mediatech, Inc., Herndon, VA, USA) supplemented with 10% fetal bovine serum (HyClone, Logan, UT, USA), and 1% penicillin/streptomycin (Mediatech, Inc.), while THP-1 cells were cultured in RPMI 1640 supplemented with 10% fetal bovine serum (HyClone, Logan, UT, USA) and 1% penicillin/streptomycin. Cells were cultured at 37°C in a water-jacketed 5% CO_2_ incubator.

### M2-TAM generation and co-culture

M2-polarized THP-1 macrophages (referred to as M2-TAMs from hereon) were generated as described previously [13]. Briefly, THP-1 cells (1×10^6^) were seeded into the upper insert of a six-well Transwell apparatus (0.4 μm pore size, Corning, Lowell, MA) and treated with 320 nM phorbol myristate acetate (PMA) for 6 h, followed by incubation with PMA and IL-4 (20 ng/ml) and IL-13 (20 ng/ml) for an additional 18 h. After a thorough wash to remove all PMA, PMA-treated M2 TAMs (upper inserts) were co-cultured with either A549 or H441 cells (in a six-well plate, 2×10^5^ cells per well) without direct contact for 48 h. Different concentrations of pterostilbene (5, 10, and 20 μM) were added 6 h after seeding A549 or H441 cells (after cells have attached to the plate). Co-cultured lung cancer cells were then washed and harvested for subsequent experiments.

### Isolation of CD133^+^ cancer stem cells using Fluorescence activated cell sorting (FACS)

CD133^+^ cells were labeled with specific antibodies or isotype control antibodies (all from Coulter- Immunotech Co., Miami, FL, USA) and sorted using magnetic microbeads (Miltenyi Biotec, Auburn, CA, USA). Cells were analyzed and sorted on a FACSAria Cell Sorter unit (Becton Dickinson), using propidium iodide (PI) as a viable stain. Cells were gated on low side scatter, low-to-moderate forward scatter, and low PI. For data acquisition, at least 10,000 events were analyzed. The purity and viability of isolated cells were routinely > 98%.

### Lung cancer sphere formation essay

Sorted single cells were plated on ultralow attachment plates (Corning, Acton, MA, USA) at a density of 20,000 viable cells/ml in cultures of sorted lung cancer cells and a density of 1000 cells/ml for passages. Cells were grown in serum-free DMEM medium, supplemented with B27 (Invitrogen, Carlsbad, CA, USA) and 10 ng/ml epidermal growth factor (BD Biosciences, Palo Alto, CA, USA). Lung cancer spheres were collected by gentle centrifugation (58 g, 800 r.p.m.) after 10 days, and were dissociated enzymatically (10 min in 0.05% trypsin) and mechanically, using a fire-polished Pasteur pipette. The cells obtained from dissociation were passed through a 40-μm sieve and analysed microscopically for single cellularity. Cells plated at low densities (1000 cells/ml) were grown in conditioned medium from high-density sorted cultures in suspension.

### Aldefluor assay

ALDH activity was detected using the Aldefluor assay kit (Stemcell Technologies, Vancouver, Canada) as described by the manufacturer. Briefly, cells were suspended in Aldefluor assay buffer containing ALDH substrate and BODIPY-aminoacetaldehyde (BAAA). The BAAA was taken up by living cells and converted by intracellular ALDH into BODIPY-aminoacetate, which yields bright fluoresce. The brightly fluorescent ALDH-expressing cells were detected with FACS Aria II (BD Biosciences, Franklin Lakes, NJ). As a negative control, cells were stained under identical conditions in the presence of the specific ALDH inhibitor, diethylaminobenzaldehyde (DEAB; Sigma). Data were analyzed by Cell Quest software (BD Biosciences).

### RNA extraction and qRT-PCR

The mRNA levels of *Muc1, Sox2, CD133, E-cadherin, VEGF and NF-κB* were quantified by quantitative reverse transcription-polymerase chain reaction (qRT-PCR), using *GAPDH* mRNA as an internal control. Briefly, both A549 and H441 in parental and its counterpart subpopulation cells were cultured in 100-mm tissue culture dishes and total RNA was extracted with a Trizol RNA isolation kit. RNA concentration and purity were determined based on measurement of the absorbance at 260 nm and 280 nm. Two micrograms of total RNA was reverse transcribed using SuperScript II Reverse Transcriptase (Invitrogen) and oligo-dT primers according to the manufacturer's instructions. Forward and reverse primers for *Muc1*, *Sox2*, *CD133*, *E-cadherin*, *VEGF*, *and NF-κB* were designed using the Primer Express 1.5 software (Applied Biosystems). Amplification reaction assays contained 1× SYBR Green PCR Mastermix (Applied Biosystems) and primers (Applied Biosystems) at the optimal concentrations. A hot start at 95°C for 5 minutes was followed by 40 cycles at 95°C for 15 seconds and 65°C for 1 minute using an ABI PRISM 7500 Real-Time PCR System (Applied Biosystems). GAPDH was used as the reference gene for normalization and the mRNA expression level was quantified using the threshold cycle method.

### Gene silencing using siRNA technique

Both A549 and H441 cells were transfected with SignalSilence^®^ MUC1 siRNA I #13253 (Cell Signaling, Danvers, MA) according to the vendor's instructions. Comparative gene expression analysis of selected M1- and M2-associated transcripts in macrophages after being treated with control siRNA or MUC1 siRNA in THP-1 cells. Transfected cells were lysed and subjected to both total RNA extraction and western blot analysis 48 hours post transfection. The MUC1 expression level was assayed using MUC1 (VU4H5) Mouse mAb #4538 (Cell Signaling, Danvers, MA).

### Western blotting

Cell lysates were prepared using ReadyPrep Protein Extraction Kit (Bio-Rad, Hercules, CA) according to the instructions provided. Total cell lysates (50μg) were separated electrophoretically by a 10% polyacrylamide SDS-PAGE gel and transferred onto a polyvinylidene fluoride membrane using the BioRad Mini Protean transfer system. The blots were then blocked with 5% skim milk in PBST for 1 h and probed with primary antibodies overnight at 4°C. All primary antibodies were purchased from Cell Signaling unless otherwise specified. The membranes were sequentially detected with an appropriate peroxidase-conjugated secondary antibody incubated at room temperature for 1 h. Blots were washed 3 times with PBS. Signals were then detected using an enhanced chemiluminescence detection system and BioSpectrum Imaging System (UVP, Upland, CA).

### Immunohistochemical staining for MUC1 and TAMs

A total of 50 patients diagnosed with lung adenocarcinoma between January 1, 2005 and December 31, 2010 in MacKay Memorial Hospital (Taipei City, Taiwan) were enrolled for the study. All of the patients gave signed, informed consent for their tissues to be used for scientific research. Recommendations of the Declaration of Helsinki for biomedical research involving human subjects were also followed. Ethical approval for the study was obtained from Joint Institutional Review Board of the Mackay Memorial Hospital (approval number: 13MMHIS082). Patients' clinical records were reviewed to determine the stage of the tumor at the time of diagnosis and outcome. The Immunoperoxidase staining for MUC1 was performed manually by a board-certified lab technician with strict adherence to the manufacturers' instructions for both technical and interpretative issues. Tissue was cut at 4 μm and mounted on positively charged glass slides. Slides were dried overnight at 60°C, deparaffinized, and then rehydrated [two changes of xylene (10 min each); two changes of 100% ethanol (3 min each); two changes of 95% ethanol (3 min each); and 2 changes of distilled water (2 min each)]. Endogenous peroxidase activity was blocked with 0.3% hydrogen peroxide for 15 min. For antigen retrieval, tissue slides were boiled in 10 mM citrate buffer (pH 6.0) and microwave-treated for 10 min. Nonspecific binding was blocked with 10% normal rabbit serum for 20 min. The tissue slides were incubated with mouse monoclonal antibody against human MUC1 (ab15481; 1:100 dilution) overnight at 4°C. After rinsing five times with 0.01 M phosphate-buffered saline (PBS; pH = 7.4) for 10 min, the detection of the primary antibody was achieved with a secondary antibody (Envision; Dako, Glostrup, Denmark) for 1 h at room temperature, and stained with DAB (30,30-diaminobenzidine) after washing in PBS again. Finally, the sections were counterstained with Mayer's hematoxylin, dehydrated, and mounted. To ensure accurate and reproducible staining, a section of gastric adenocarcinoma was used as a positive control, while phosphate buffered saline replaced anti-MUC1 antibody as a negative control. In addition, immunohistochemistry for TAMs was carried out on consecutive sections using a three step protocol with a monoclonal mouse anti-CD68 antibody (1:100 dilution; Abcam, UK) as the primary antibody and a rabbit anti-mouse IgG antibody conjugated to horseradish peroxidase (ZSGB-BIO) as the secondary antibody. MUC1 and CD68 staining in cancer cells and TAMs were evaluated by authorized pathologists who had no knowledge of the patient clinical status and outcome.

### Statistical analysis

Each experiment was performed in triplicate. The analysis of variance (ANOVA) was conducted to evaluate the effects of pterostilbene on cell viability. Following a significant ANOVA, a post-hoc comparison using the Bonferroni adjustment was applied. The significant difference between control and experimental groups was analyzed using Student's *t*-test. (*, *p* < 0.05; **, *p* < 0.01).

## SUPPLEMENTARY FIGURES AND TABLES


