# Supplementary material for: Modulation of macrophage polarization and lung cancer cell stemness by MUC1 and development of a related small-molecule inhibitor pterostilbene
Source: Oncotarget. 2016 Jun 4;7(26):39363–75. doi: 10.18632/oncotarget.8101 (PMC5129938; doi:10.18632/oncotarget.8101)
Supplement: Supplementary file 1 [file oncotarget-07-39363-s001.pdf]

**Modulation of macrophage polarization and lung cancer cell stemness by MUC1 and development of a related small-molecule inhibitor pterostilbene**

**Supplementary Material**

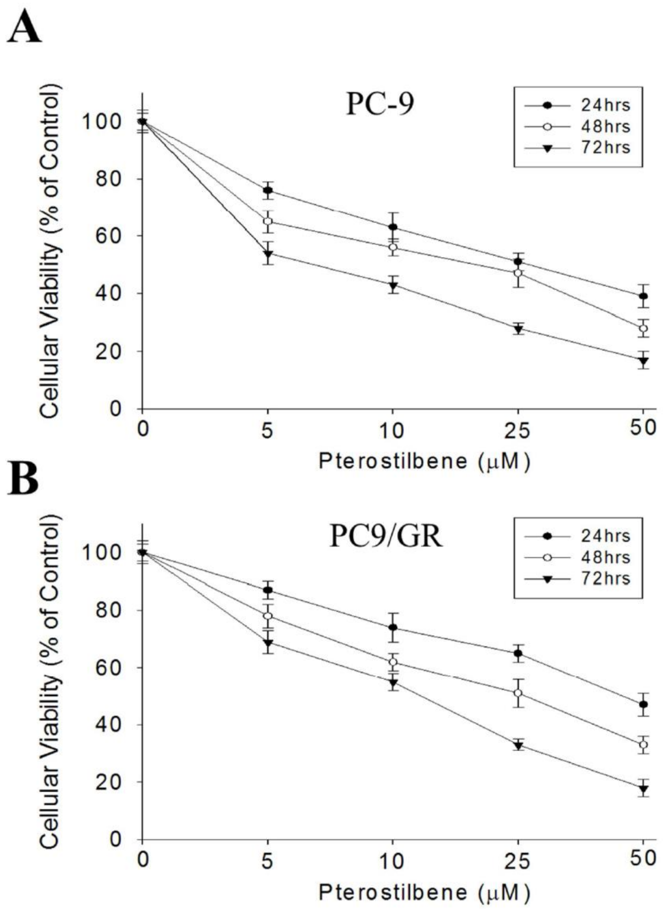


**Supplemental Figure S1.** MTT assays were used to examine the inhibitory activities of pterostilbene on lung cancer cell proliferation. The cells were exposed to various concentrations of pterostilbene in PC-9 (EGFR exon 19 in-frame deletion) and PC-9/GR (gefitinib-acquired resistant cells) cells for 72 h. Each data point is the result of more than three independent experiments.

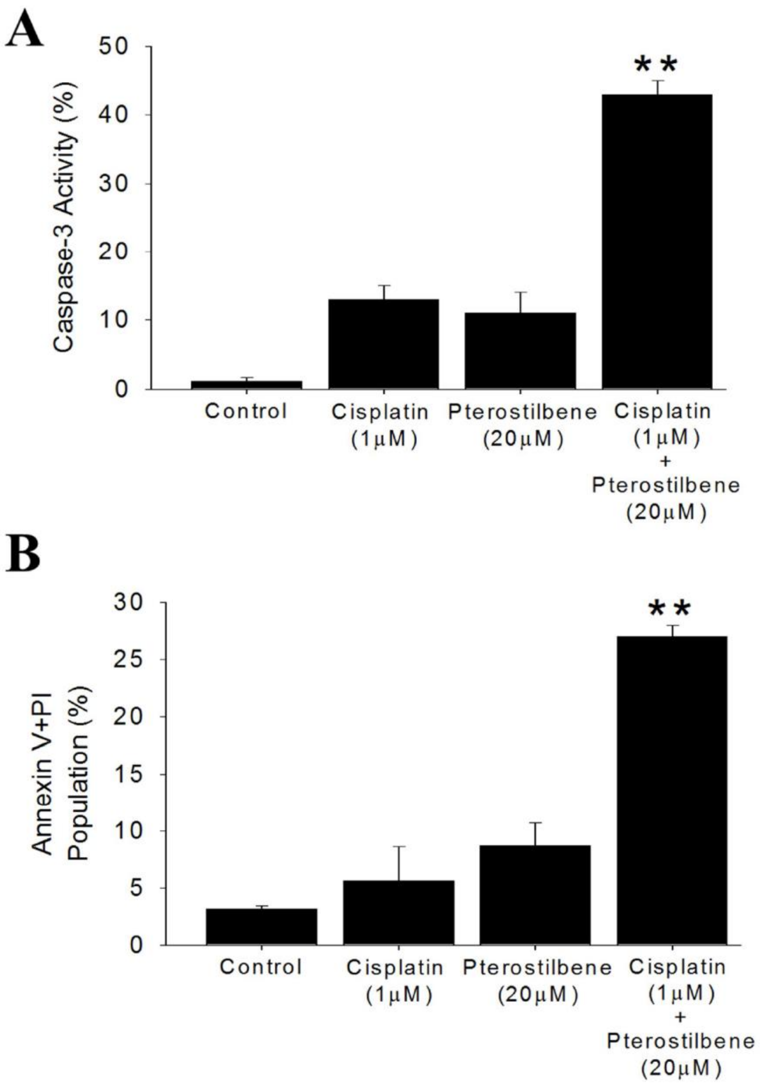


**Supplementary Figure S2.** Synergistic apoptosis analysis of the combination treatment. Both Caspase-3 activity (A) and Annexin V-PI conjugates for apoptosis (B) were measured in H441 cells treated with DMSO as the vehicle control, cisplatin (1μM), pterostilbene (20 μM), and the combination. * $P < 0.05$ when compared to the control (DMSO).
